# Seed Priming with Melatonin Improves the Seed Germination of Waxy Maize under Chilling Stress via Promoting the Antioxidant System and Starch Metabolism

**DOI:** 10.1038/s41598-019-51122-y

**Published:** 2019-10-21

**Authors:** Qingjun Cao, Gang Li, Zhengguo Cui, Fentuan Yang, Xiaoli Jiang, Lamine Diallo, Fanli Kong

**Affiliations:** 1 Jilin Academy of Agriculture Science/Key Laboratory of Northeast Crop Physiology Ecology and Cultivation, Ministry of Agriculture and Rural Affairs of the People’s Republic of China, Changchun, 130033 P.R. China; 2 College of Plant Science, Jilin University, Changchun, 130062 P.R. China

**Keywords:** Plant physiology, Abiotic

## Abstract

Chilling stress is one of the major abiotic stresses affecting waxy maize plant growth. Melatonin (MT) is able to improve tolerance to abiotic stress in plants. To investigate the effects of seed priming with MT on tolerance to chilling stress in waxy maize, the seed germination characteristics and physiological parameters were tested with varied MT concentrations (0, 50, 100 µM) and treatment times (12, 24 h) at ambient (25 °C) and chilling (13 °C) temperature. MT primed seeds significantly enhanced the germination potential (by 20.29% and 50.71%, respectively), germination rate (by 20.88% and 33.72%), and increased the radicle length (by 90.73% and 217.14%), hypocotyl length (by 60.28% and 136.14%), root length (by 74.59% and 108.70%), and seed vigor index (46.13%, 63.81%), compared with the non-priming seeds under chilling stress. No significant difference was found in priming time between primed and non-primed seeds. In addition, lower H_2_O_2_ and malondialdehyde concentrations, increased antioxidant enzyme activities (superoxide dismutase, peroxidase, catalase and ascorbateperoxidase), and promoted starch metabolism were found in primed seeds compared to non-primed ones. It was suggested that seed priming with MT improved waxy maize seed germination under chilling stress through improving antioxidant system and starch metabolism, which protected from oxidative damage.

## Introduction

Chilling stress (CS) is a critical factor that determines the geographical distribution of many field crop species and their productivity^1^. Waxy corn is extensively planted in China and in many other countries due to its high starch content. However, it is a thermophilic crop species and, as such, is highly sensitive to low temperature during germination and early seedling establishment^2,3^. CS during seed germination causes severe problems around the world including in China^4^, the United States, South Korea, Poland^5^ and Switzerland^6^. Moreover, due to global climate change, increased frequencies of extreme weather events, such as unexpected and sudden chilling events occurring after seed sowing often have negative effects on maize seed germination and shoot growth in northeastern China.

Seed germination is a complicated physiological process involving the absorption of water, the degradation of storage substances, seed respiration for energy metabolism, transcript (mRNA) synthesis and mitochondrial repair and multiplication^7^. CS thermodynamically constrains the kinetics of many physiological and metabolic processes in plants. It has been widely reported that the deleterious effects of chilling stress on crops are caused by the induction of oxidative stress. CS leads to the generation of large amounts of reactive oxygen species (ROS) including O_2_^−^, HO_2_^−^and H_2_O_2_ in plant cells and triggers lipid peroxidation reactions within membranes^6^. The excessive production of ROS jeopardizes critical cellular and metabolic functions and causes significant damage to proteins, lipids, carbohydrates and DNA, which can ultimately lead to cell death. Therefore, it is important to protect maize seedlings from chilling stress.

In recent years, various strategies have been employed to enhance abiotic stress tolerance in plants^1^. Seed priming as a pre-sowing seed treatment has been proven to be an approach for achieving rapid and uniform emergence effectively, practically and easily as well as for improving seed vigour and seedling viability under unfavourable environmental conditions^6,8^.

Melatonin (N-acetyl-5-methoxytryptamine(MT)), an indoleamine, is a highly conserved molecule occurring in evolutionarily distant organisms and has proven to be an abiotic antistress agent in plants^9–11^. As a pleiotropic molecule with various functions in animals and plants, MT has been shown to be an efficient growth regulator in plants^12,13^. Several studies have demonstrated that, lower dose of MT ( < 10 μM) can stimulate the growth of maize plants and the germination of cucumber seeds under cold stress^14–16^. In cucumber, seed priming with MT promotes lateral root formation and seed germination under water-stress conditions^17^. In addition, MT acts as the first-line of defence and as an internal sensor of oxidative stress in plants. For instance, exogenous applications of MT can increase photosynthetic C assimilation by improving plant antioxidant defence of organelles under drought or low temperature stress in barely^9^. MT can also increase the chance of survival by enhancing starch metabolism and energy supplies in response to damage caused by environmental stressors from heavy metals and temperature fluctuations^18,19^. Thus, improved performance of primed seeds in terms of germination and seedling growth under chilling stress might be attributed to an enhanced antioxidant system and increased starch metabolism. However, information regarding the effects of waxy corn seed priming with MT in plants in response to chilling stress is limited.

Therefore, the objectives of this study were to investigate the effects of seed priming with MT on CS mitigation during waxy corn germination. We hypothesized that the protective role of MT was related to 1) enhanced antioxidant capability, 2) osmotic regulation, such as the accumulation of soluble sugars or protein, and 3) increased sugar metabolism and activities of related enzymes involved in carbohydrate metabolism in germinating seeds under CS.

## Results

### Effects of MT priming on seed germination rates

**Figure 1:**
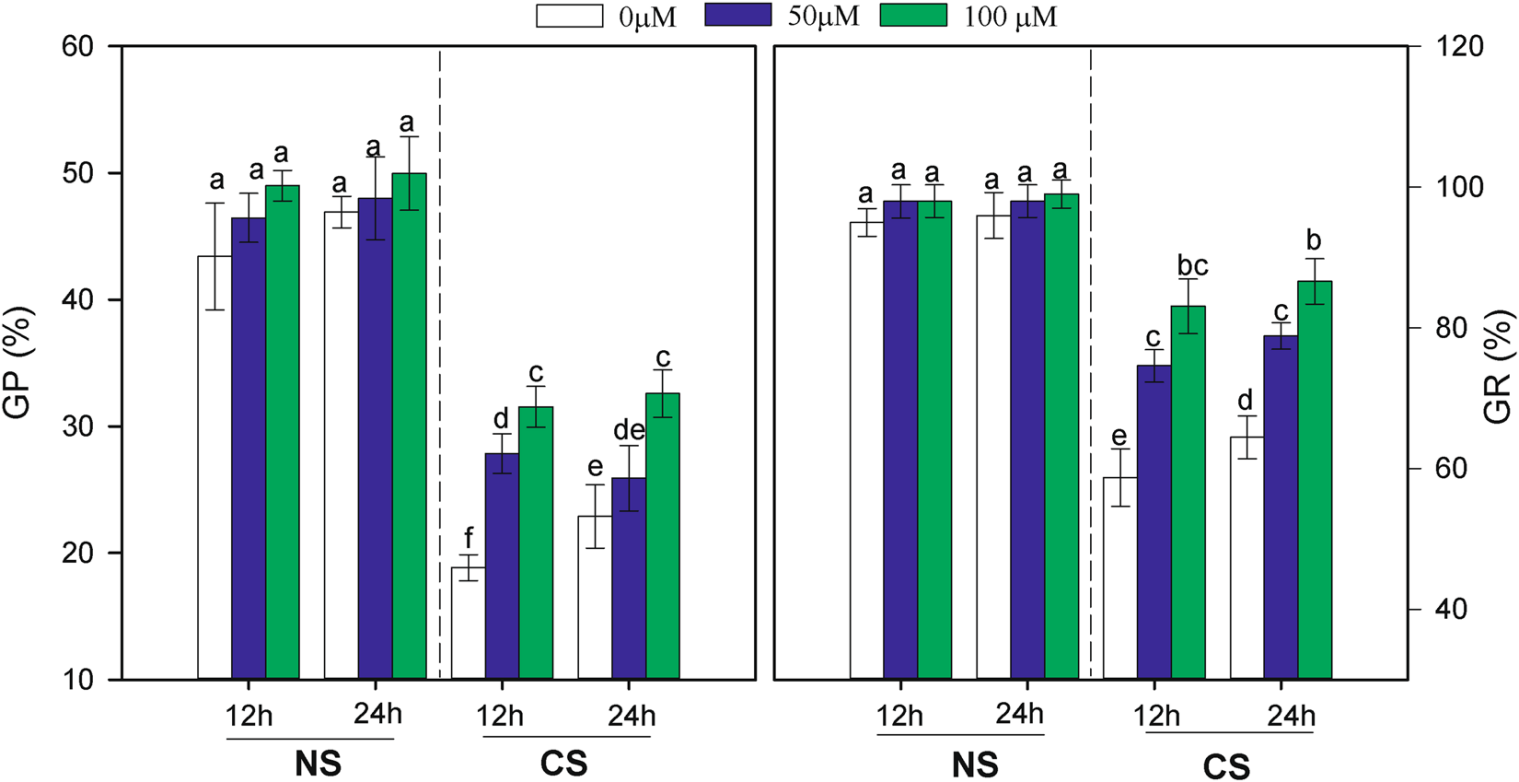
Effects of melatonin priming on the germination potential (GP) and germination rate (GR) of waxy corn seeds under non-chilling stress (NS) and chilling stress (CS). The seeds were germinated with variouss of melatonin (0, 50 and 100 µM) under priming times of 12 and 24 h. The **v**ertical bars represent the mean values ± S.D. of three replicates (n = 3); the different lowercases indicate different significances at 0.05 level. The terms 12 h and 24 h represent seed priming times for 12 h and 24 h, respectively.

Seed priming and germination temperature significantly affected the potential (GP) and germination rate (GR) (Fig. 1). CS significantly decreased the GP and GR of MT-primed and MT-non-primed seeds. As shown in Fig. 1, seeds primed with MT at 50 and 100 µM under CS had a higher GP and GR than did the control seeds (non-primed treatment) under both priming time treatments (12 and 24 h). However, the GP and GR were not significantly affected by priming time (*P* > 0.05) under either the CS or the non-chilling stress (NS) treatments.

### Effects of MT priming on seed germination characteristics

**Figure 2:**
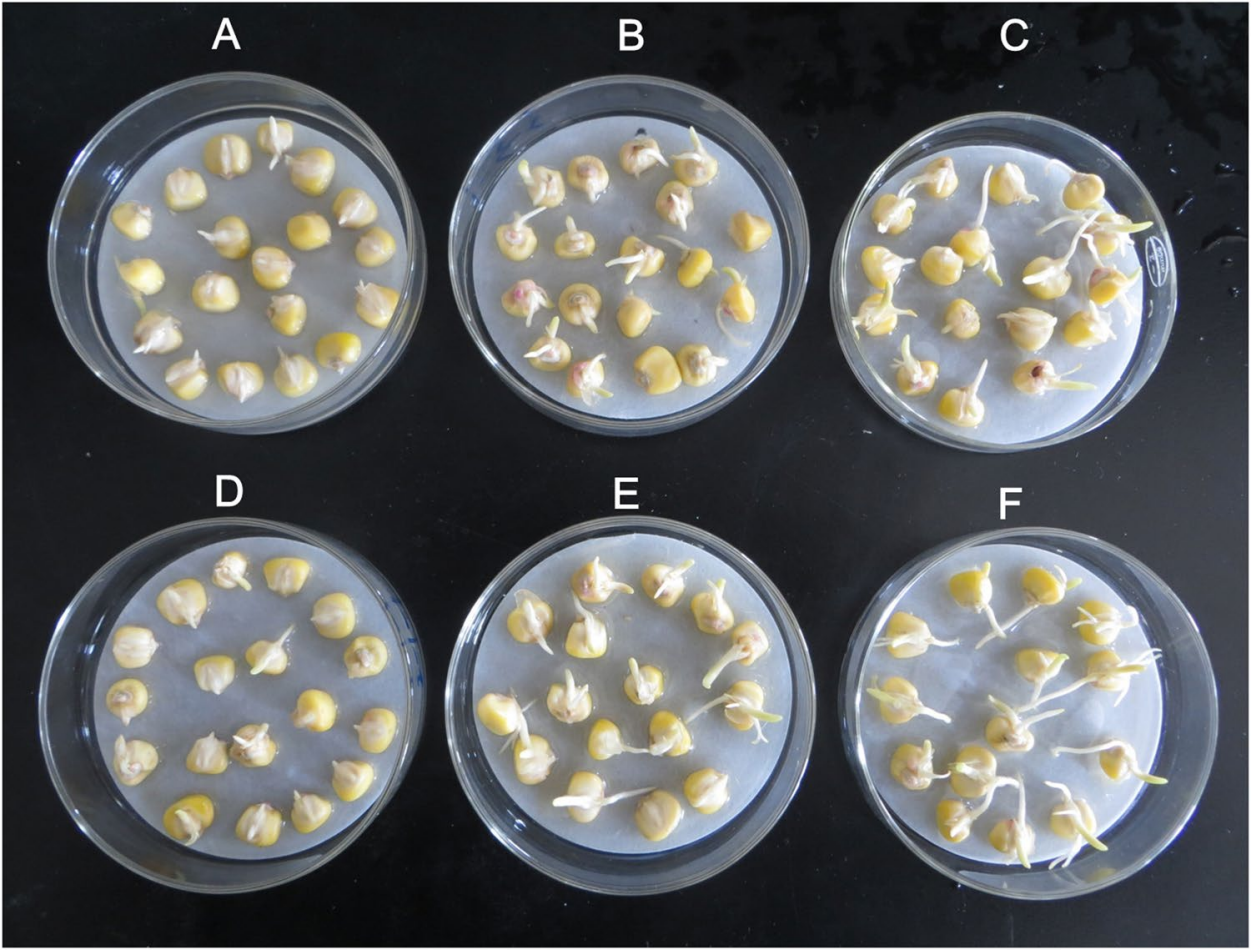
Seeding morphology characteristics after 9 days germinated with various concentrations of melatonin: (**A**) 0 µM, (**B**)50 µM and (**C**)100 µM priming for 12 h, and (**D**) 0 µM, (**E**) 50 µM and (**F**)100 µM for 24 h, correspondingly.

Seed germination characteristics including radicle length (RAL), hypocotyl length (HL), root length (RL), germination index (GI) and seed vigour index (VI) were significantly affected by germination temperature and MT priming (Table 1 and Fig. 2). As shown in Table 1, marked reductions in RAL, HL, RL, the GI and the VI were observed for primed and non-primed seeds under CS treatment. However, compared with non-primed seeds, seeds primed with MT on the dose 50 and 100 µM exhibited significantly enhanced seed germination characteristics including RAL, HL, RL, GI and VI under CS and VI under NS treatment (Table 1), while no significant differences were found between priming time treatment (12 h and 24 h). In addition, the GI was significantly affected by the interaction (T x C) of germination temperature (T) and MT concentration (C), and the VI was significantly affected by the interaction (T x PT) of germination temperature (T) and seed priming time (PT) (Table 1).

**Table 1:** Effects of seed priming with melatonin (MT) on seed germination characteristics in waxy corn on the 9th day germination under NS and CS condition, with varied priming times (12 h and 24 h).

Temperature	Priming Time(h)	MT concentration (µM)	RAL	HL	RL	GI	VI
NS	12 h	0	46.70 ± 5.5a	37.11 ± 3.6a	41.98 ± 2.5a	20.78 ± 0.49a	5.97 ± 0.46c
		50	54.18 ± 3.7a	41.67 ± 5.8a	43.2 ± 3.9a	22.08 ± 0.56a	7.63 ± 0.75b
		100	56.84 ± 6.3a	45.44 ± 6.7a	46.33 ± 2.9a	21.97 ± 0.84a	8.83 ± 0.44b
	24 h	0	48.01 ± 3.5a	36.95 ± 5.43a	40.85 ± 3.8a	20.97 ± 1.07a	7.61 ± 0.58b
		50	52.71 ± 4.6a	40.83 ± 3.64a	42.25 ± 3.3a	21.30 ± 2.75a	8.13 ± 0.51b
		100	53.45 ± 2.1a	43.92 ± 3.62a	43.4 ± 2.03a	22.92 ± 0.80a	9.63 ± 0.77a
CS	12 h	0	5.29 ± 1.3e	6.29 ± 1.8d	5.57 ± 1.7c	8.49 ± 1.18c	3.02 ± 0.47d
		50	13.13 ± 4.1d	10.75 ± 2.7c	11.08 ± 2.06b	11.08 ± 1.02b	4.06 ± 1.08a
		100	20.75 ± 3.2c	13.25 ± 3.5c	12.75 ± 3.13b	12.29 ± 1.56b	5.05 ± 0.49c
	24 h	0	6.88 ± 0.64e	6.57 ± 2.30d	7.10 ± 0.69c	8.51 ± 0.61c	3.21 ± 0.47d
		50	14.40 ± 3.78d	9.33 ± 4.76c	11.80 ± 2.97b	12.80 ± 0.54b	5.04 ± 0.50c
		100	19.60 ± 2.55c	13.40 ± 2.8c	12.40 ± 2.20b	14.00 ± 0.92b	5.15 ± 0.49c
**Source of variation**							
Temperature, T	***	***	***	**	***		
Priming Time, PT	ns	ns	ns	ns	ns		
MT concentration, C	***	***	***	**	***		
T × PT	ns	ns	ns	ns	*		
T × C	ns	ns	ns	**	ns		
PT × C	ns	ns	ns	ns	ns		
T × PT × C	ns	ns	ns	ns	ns		

Values are mean ± S.D. (n = 4). Different letters in a vertical column indicate a significant difference between each treatment under different MT concentration. NS = non-chilling stress; CS = chilling stress; RAL = radicle length; HL = hypocotyl length; RL = root length; GI = germination index; VI = seed vigour index. The *, ** and *** indicate a significant difference existing at 0.05, 0.01 and 0.001, respectively.

### Effects of MT priming on H_2_O_2_ and malondialdehyde production

**Figure 3:**
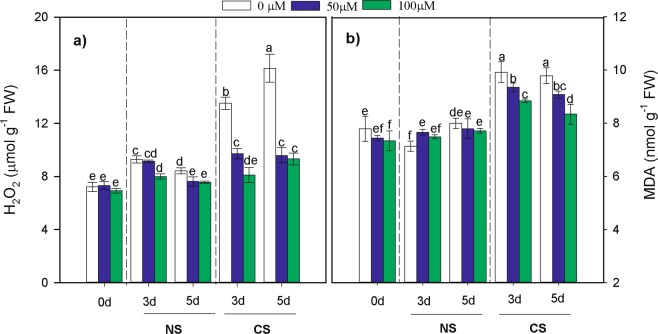
Effects of melatonin priming on (**a**) H_2_O_2_ and (**b**) MDA content of waxy corn seed under non-chilling stress (NS) and chilling stress (CS) on the 0th, 3th, 5th day of germination. The seeds were germinated with various concentrations of melatonin (0, 50 and 100 µM) with priming time of 12 h.Vertical bars represent means value ± S.D. of three replicates (n = 3); Different lowercases indicate difference significant at 0.05 level.

The dynamic changes in H_2_O_2_ and malondialdehyde (MDA) contents are shown in Fig. 3. In non-primed corn seeds (control), exposure to CS conditions resulted in significantly higher H_2_O_2_ and MDA contents compared with those in primed seeds for both priming (12 h and 24 h), while seed priming with MT significantly decreased the H_2_O_2_ and MDA contents in germinated seeds under both CS and NS treatments (Table 2). Compared to those in non-primed seeds, H_2_O_2_ and MDA contents in primed seeds under CS treatment were reduced by 34.11% and 8.48% on the 3rd day; and by 41.44% and 11.90% on the 5th day, respectively. Moreover, seed priming with MT at a concentration of 100 µM was the more effective at reducing H_2_O_2_ and MDA contents in both 12 and 24 priming treatments.

**Table 2:** Output of Three-way ANOVA for the effects of germination temperature (T), MT concentration(C), germination days (D) and their interaction on the physiological parameters of maize seedlings.

	T	C	D	T × C	T × D	C × D	T × C × D
H_2_O_2_	***	***	ns	***	***	***	*
MDA	***	***	ns	***	***	ns	ns
Soluble protein	*	***	***	ns	**	***	ns
SOD	***	***	***	**	**	ns	ns
POD	**	***	***	**	***	ns	ns
CAT	***	***	ns	***	***	ns	ns
APX	*	***	***	ns	**	***	ns
Starch	***	**	***	ns	***	***	***
Sucrose	***	ns	***	***	*	ns	ns
Reducing sugar	*	***	**	ns	ns	**	ns
Total soluble sugar	ns	***	**	ns	ns	**	ns
α-amylase	***	*	ns	*	*	ns	ns
β-amylase	***	**	***	**	ns	ns	ns
SUS	***	***	***	**	ns	ns	ns

The *, ** and *** indicate a significant difference existing at 0.05, 0.01 and 0.001, respectively.

### Effects of MT priming on soluble protein contents

**Figure 4:**
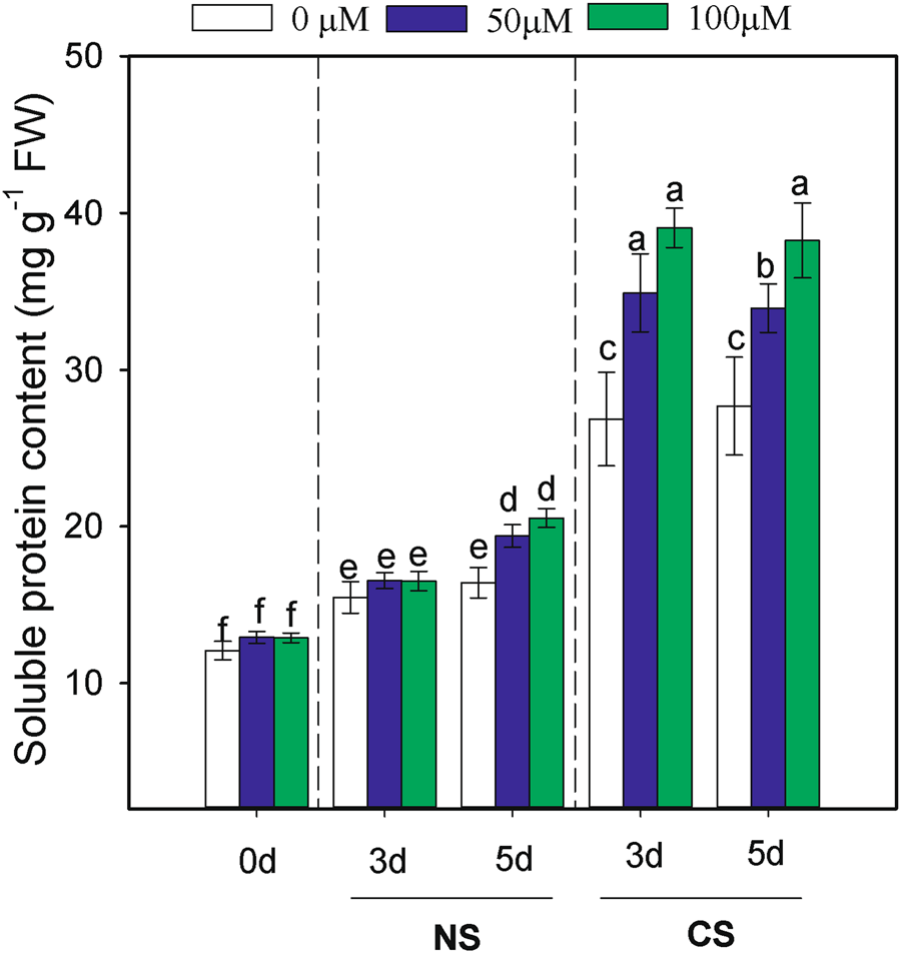
Effects of melatonin priming on soluble protein content of waxy corn seed under non-chilling stress (NS) and chilling stress (CS) on the 0th, 3th, 5th day of germination. The seeds were germinated with various concentrations of melatonin (0, 50 and 100 µM) with priming time of 12 h. Vertical bars represent means value ± S.D. of three replicates (n = 3); Different lowercases indicate difference significant at 0.05 level.

The dynamic changes in soluble protein contents are shown in Fig. 4. Seed priming and CS significantly affected the contents of soluble protein in the germinated seeds (Table 2). Higher soluble protein content was detected in MT-primed seeds compared with non-primed seeds under CS treatment. Compared to those in non-primed seeds under CS, the soluble protein contents in MT-primed seeds under CS increased by 45.46% and 29.99% on the 3rd day, and by 38.14% and 22.60% on the 5th day.

### Effects of MT priming on antioxidant enzyme activities

**Figure 5:**
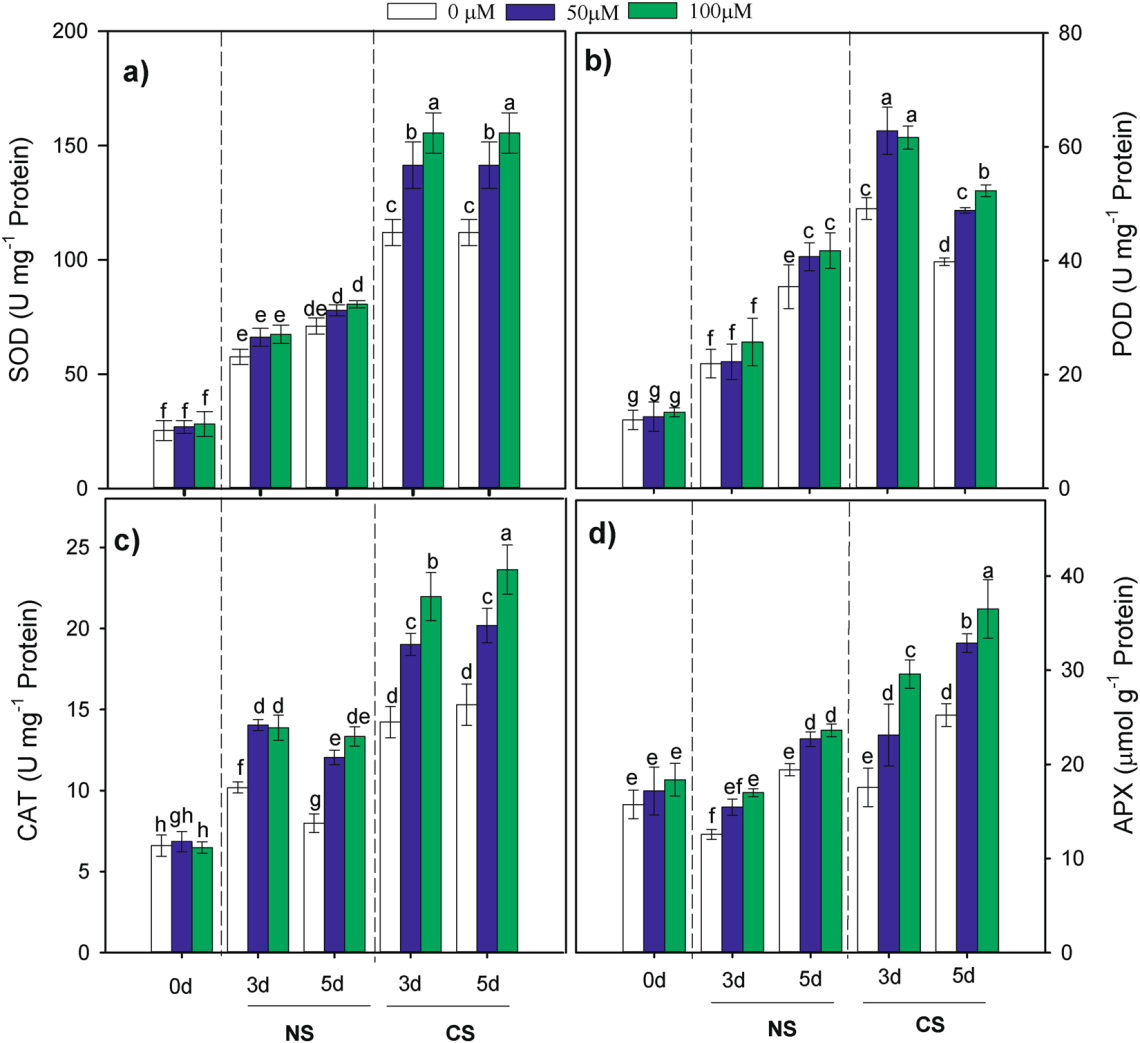
Effects of melatonin priming on the activity of (**a**) SOD, (**b**) POD, (**c**) CAT and (**d**) APX of waxy corn seed non-chilling stress (NS) and chilling stress (CS) on the 0th, 3th, 5th day of germination. The seeds were germinated with various concentrations of melatonin (0, 50 and 100 µM) with priming time of 12 h.Vertical bars represent means value ± S.D. of three replicates (n = 3); Different lowercases indicate difference significant at 0.05 level.

To confirm the high activity of the antioxidative system induced by MT, we investigated the dynamic changes in the antioxidant enzymes including superoxide dismutase (SOD), peroxidase (POD), catalase (CAT) and ascorbateperoxidase (APX) in maize seedlings. At 0, 3 and 5 days of seed germination, the activity of the antioxidant enzymes (SOD, POD, CAT and APX) in MT-primed seeds under CS and NS treatment are presented in Fig. 5. Compared with the NS treatment, seed priming with MT under CS had significant stimulatory effects on the SOD, POD, CAT and APX activities during seed germination (Table 2). In response to CS, SOD and POD activities increased rapidly and then peaked on the 3rd day of germination (Fig. 5a,b), while the CAT and APX activities showed a gradually increasing trend during seed germination (Fig. 5c,d). The SOD, POD CAT and APX activities were enhanced by 406.98%, 356.78%, 204.08% and 84.52% during the 3rd day of germination, respectively, and by 376.51%, 270.85%, 183.93%, and 37.02% on average during the 5th day of germination, respectively.

### Effects of MT priming on carbohydrate metabolism during germination under chilling stress

**Figure 6:**
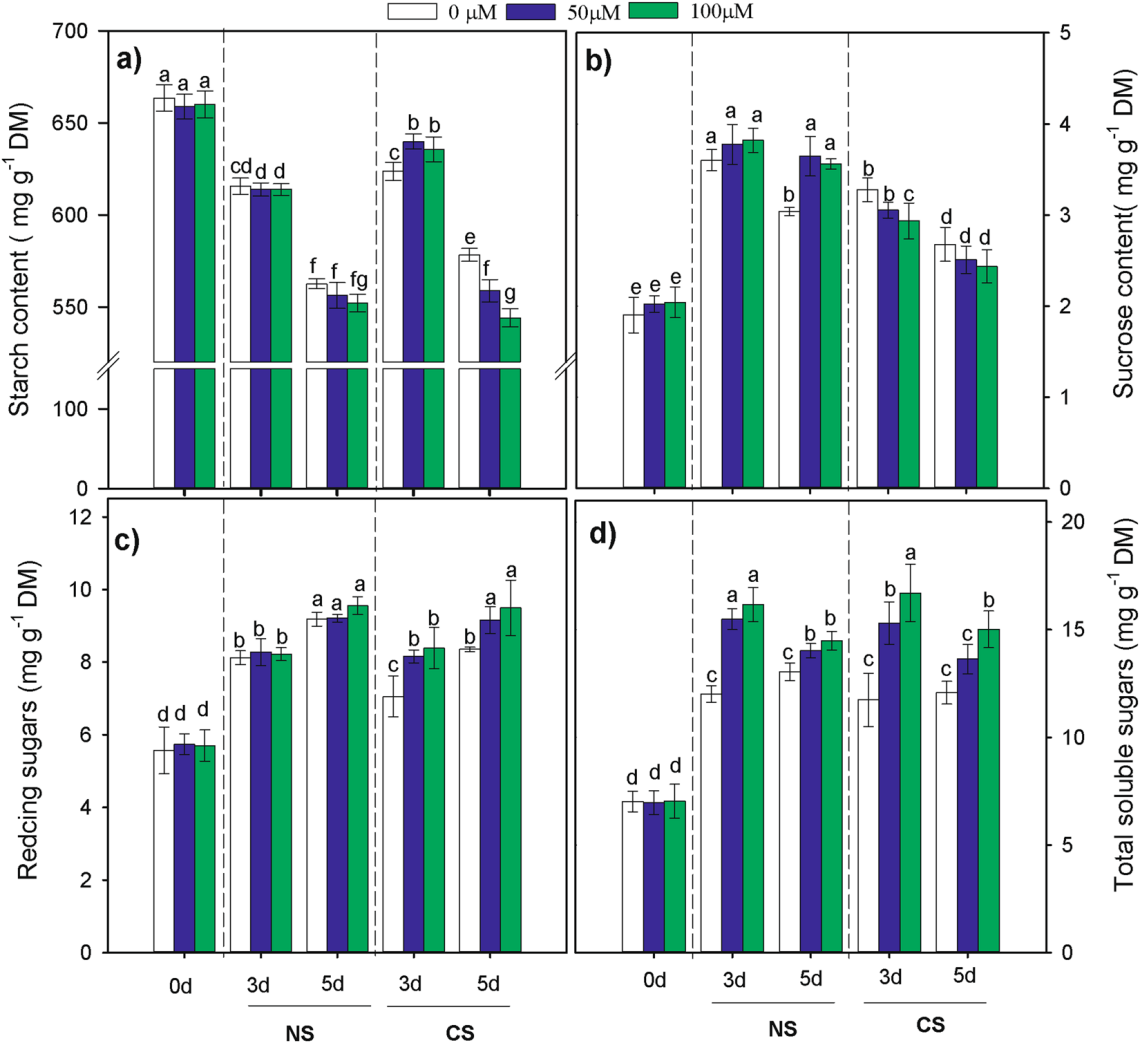
Effects of melatonin priming on (**a**) starch, (**b**) sucrose, (**c**) reducing sugar and (**d**) total soluble sugar content of waxy corn seed under non-chilling (NS) and chilling stress (CS) on the 0th, 3th, 5th day of germination. The seeds were germinated with various concentrations of melatonin (0, 50 and 100 µM) with priming time of 12 h. Vertical bars represent mean value ± S.D. of three replicates (n = 3); Different lowercases indicate difference significant at 0.05 level.

Sugar metabolism in corn seedlings was assessed in terms of starch, total soluble sugar, reducing sugar and sucrose contents during seed germination; the results are shown in Fig. 6. Temporal data during the 0, 3, and 5 days of seed germination revealed that there were significant (*P* ≤ 0.05) variations in sugar metabolism affected by CS and the seed priming treatment. From the beginning (0 d) to the 3^rd^ and 5^th^ days of seed germination, an obvious decrease in starch content and an abrupt increase in sucrose and reducing sugar contents in all germinated seeds were detected under both the NS and CS treatments. Compared to that NS treatment, the starch content in the seeds under the CS treatment was higher and sucrose, soluble sugar and reducing sugar contents were lower. However, compared with non-priming treatment, seed priming with MT (at both 50 and 100 µM) significantly (*P* ≤ 0.05) enhanced the contents of total soluble sugars, reducing sugars and sucrose and reduced the starch content under CS. Compared with that in the non-primed seeds, the starch content of seeds under the seed priming treatment markedly declined, while the contents of total soluble sugars and sucrose obviously increased first but then decreased during seed germination (Fig. 6c,d), except for the reducing sugar content on the 5^th^ day of germination (Table 2).

**Figure 7:**
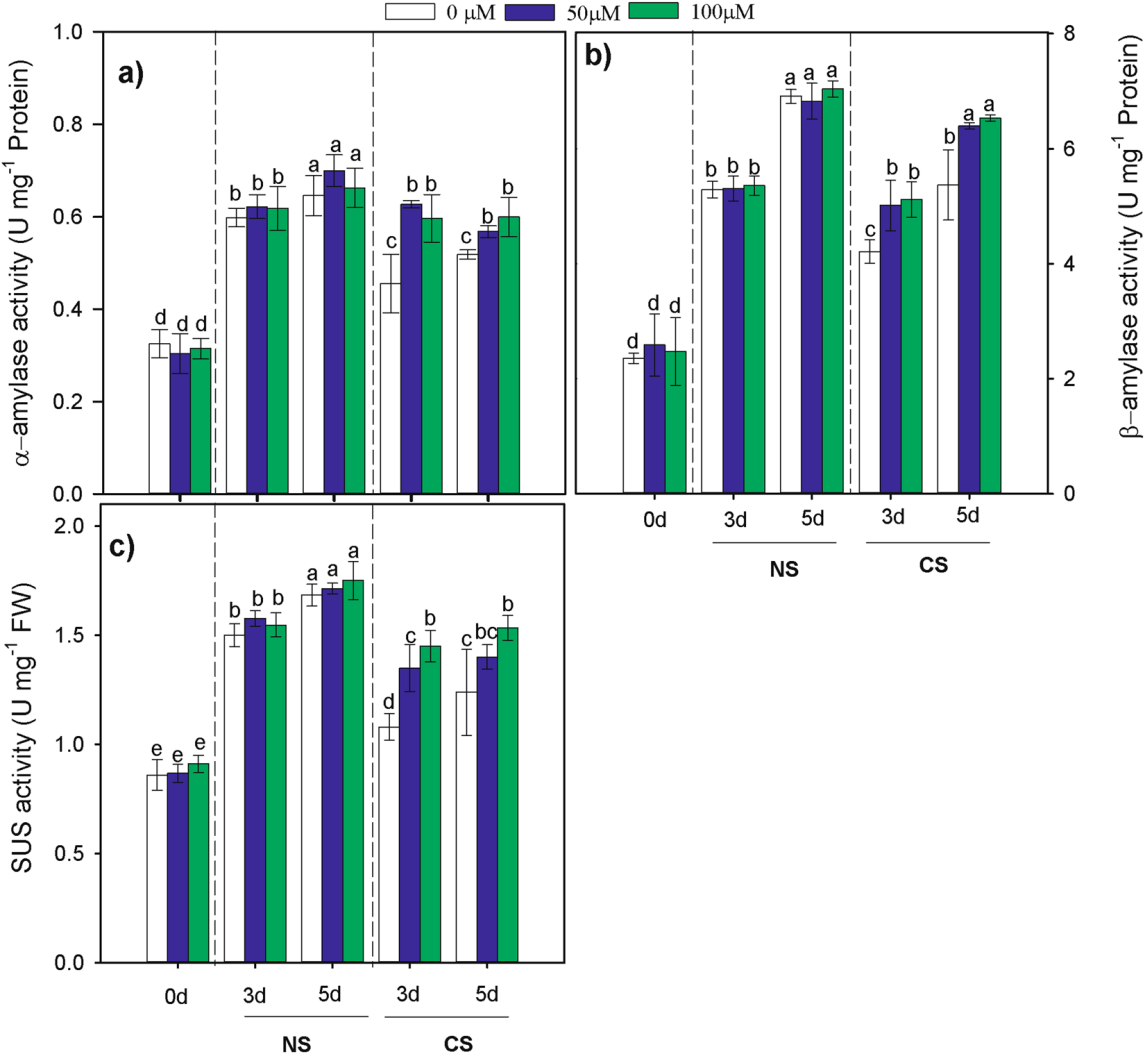
Effects of melatonin priming on (**a**) α-amylase, (**b**) β-amylase, and (**c**) sucrose synthase(SUS) activity of waxy corn seed under non-chilling (NS) and chilling stress (CS) on the 0th, 3th, 5th day of germination. The seeds were germinated with various concentration of melatonin (0, 50 and 100 µM) with priming time of 12 h.Vertical bars represent mean value ± S.D. of three replicates (n = 3); Different lowercases indicate difference significant at 0.05 level.

Seed priming with MT and CS significantly affected the activities of α-amylase, β-amylase, and sucrose synthetase (SUS) during seed germination (Fig. 7). At 0 days of germination, seed α-amylase activity, β-amylase activity, and SUS activity were similar, without statistically significant differences. While MT-primed seeds showed higher α-amylase activity, β-amylase activity, and SUS activity compared with non-primed seeds during the 3rd and 5th day of germination under CS condition on, and the α-amylase activity of MT primed seed was enhanced by 34.46% and 53.67% on the 3th and 5th day of germination under CS (Fig. 7a), respectively; moreover, the β-amylase activity increased by 20.58% and 25.08%, respectively (Fig. 7b), and the SUS activity increased by 29.62% and 18.28%, respectively (Fig. 7c). Although the activities of α-amylase, β-amylase and SUS showed an increased trend on the 3rd and 5th days of germination, but they were not significant (*P* > 0.05) in the NS treatment.

## Discussion

Seed germination is a complicated physiological and biochemical process modulated by plant growth regulators and phytohormones such as polyamines^20^, gibberellins^4^, abscisic acid and salicylic acid^21,22^. Seed priming with or exogenous applications of these agents have been proven to alleviate the adverse effects of drought and chilling stress. The results of our study suggested that seeds primed with MT not only accelerated seed germination rate but also significantly enhanced seed vigour, as indicated by longer radicle lengths, hypocotyl lengths, and root lengths compared with those of the control (Table 1). Our findings are similar to previous results^10^, in which it was demonstrated that exogenous applications of 100 µM MT have a stimulatory effect on root growth due to raising the endogenous levels of free IAA in the roots of young seedlings of *Brassica juncea*. Simlat *et al*.^16^ found that a low dose of MT (5 and 20 µM) significantly improved the seed germination and properties of *Stevia rebaudiana* Bertoni plantlet, while treatments with a higher dose showed an obvious inhibitory effect. Furthermore, Li *et al*.^9^ shown that exogenous applications of MT resulted in increased ABA concentrations and caused an alleviating effect in drought-primed plants when exposed to cold stress.

Generally, CS leads to the generation of a large amount of reactive oxygen species (ROS) including O^2−^, HO^2−^, and H_2_O_2_ in plant cells as well as elevated levels of MDA by increasing lipid peroxidation^6,8^. Recently, many studies have reported that MT plays a vital role in counteracting the effects of ROS and reducing levels of ROS in response to various stresses. For instance, exogenous MT inhibited the accumulation of O^2−^ and H_2_O_2_ in tomato seedlings and wheat leaves under chilling stress^19,23^. In the present study, exposure of non-primed waxy corn seedlings to chilling stress significantly increased the H_2_O_2_ and MDA contents, while seed priming with MT decreased the H_2_O_2_ and MDA contents (Fig. 3). This outcome demonstrated that MT played a crucial role in cold resistance in waxy corn seed germination. H_2_O_2_ and its derivatives are known to be very harmful for seed germination and seeding establishment. The H_2_O_2_ content is known to be related to endosperm cap weakening and embryo elongation during lettuce seed germination^24^. Furthermore, corn seeds with 100 µM MT were observed to be more effective at reducing H_2_O_2_ and MDA accumulation than were non-primed seeds; to some extent, the deceased levels of H_2_O_2_ and MDA in the present study might be due to the seed membrane and organelle after priming having been repaired in advance^25^.Thus, MT could act as a highly effective factor that protects plant cells from oxidative damage; this protective effect induced by melatonin was also observed in the germination of tomato seeds under drought tolerance^19^, as well as in chickpea^26^ and *Bermudagrass*^27^ under cold stress.

At the beginning of seed germination, along with the absorption of water, a series of complex physical and chemical processes occurring in germinated seeds, including the activation of various enzymatic systems, membrane repair activities and the degradation of storage substances^7^. Thus, the levels of soluble proteins and other small molecular products generally increase and provide energy, nutrients and proteins specific for seedling growth (eg., cell membrane transport protein, cold-responsive proteins) during the processes involved in the decomposition of seed storage proteins^28^. In addition, soluble proteins could also act as important osmotic regulatory substances in adverse growth environments^29^. As a defence strategy, the increase in and accumulation of soluble proteins can improve the water-holding capacity of cells; and can play a protective role for cell membranes and vital biological materials^30^. In the present study, the relatively high soluble protein accumulation after seed priming was observed (Fig. 4). Thus, the relatively high soluble protein concentration in primed waxy corn seedlings may provide protection to stabilize membranes from damage associated with functions involving membrane formation and repair under low temperature stress.

Seed priming with MT enhanced the antioxidative capability of waxy corn seedlings subjected to CS. It has been demonstrated in many crop species that SOD, POD, CAT, and APX provide protection against oxidative stress induced by chilling^2,31^. Melatonin is an antioxidant that directly and indirectly scavenges radicals that exist extensively in animals and plants^11,12,23^. The results of our study revealed that all the seeds with priming treatments had stimulatory effects on SOD, POD, CAT and APX activities during germination under CS (Fig. 5). Interestingly, the present findings showed that the SOD and POD activities increased rapidly but then peaked on the 3rd day of germination in response to chilling stress, whereas the CAT and APX activities showed a gradually increasing trend during seed germination. These results may be due to the sudden drastic reduction in the temperature at seeding, which could have induced the excessive generation of ROS under chilling stress followed by the scavenging of ROS, leading to the improvement of antioxidant enzyme activities, such as those of POD and SOD in seeds. Several other studies have confirmed that seed priming triggers increases in SOD, POD, and CAT activities during the seedling stage of plants^8,31^. These results suggest that seeds primed with exogenous melatonin can stimulate antioxidant enzyme activities in waxy corn seeding under cold conditions and thus increase the antioxidant capability under CS.

Sugar metabolism during germination and early seedling growth plays a crucial role in determining seedling vigour, particularly under stress conditions^1^. It is well documented that carbohydrates are necessary for living cells for the provision of an essential carbon source and that their carbon skeletons are used for different biosynthetic processes. Environmental stresses generally lead to major alterations in carbohydrate metabolism, and sugar signalling pathways interact with stress pathways and hence affect the expression of various genes by down- and upregulating their expression^32^. Generally, seed germination is a complex physiological process involving the degradation of storage substances and the generation of small molecular substances such as sucrose and glucose. In the present study, the starch content in response to the seed priming treatment decreased markedly, while the total soluble sugars and sucrose increased first but then decreased during seed germination (Fig. 6), as did the reduced sugars contents. These results revealed that, seed priming with MT resulted in a higher starch metabolism in seeds under chilling stress, thus ensuring a food supply to young germinating seedlings and maintaining turgor pressure for the expansion of tissues during seed germination. These results are consistent with those of studies of chickpea^26^ and *Nicotiana tabacum* L.^32^ plant cells. α-amylase, β-amylase and SUS are the key enzymes involved in the process of starch decomposition, affecting seed germination and seedling growth^26^. The results of our study suggested, compared with non-primed seeds, MT-primed seeds showed higher α-amylase and β-amylase activities, and enhanced SUS activity (Fig. 7), which were consistent with the reduction in starch content and elevated sucrose levels (Fig. 6b) in germinating seeds. These findings are similar to those obtained by Farooq, M. *et al*.^26^, who demonstrated that disrupted sugar metabolism under CS may be due to changes in α-amylase activity.

## Conclusion

**Figure 8:**
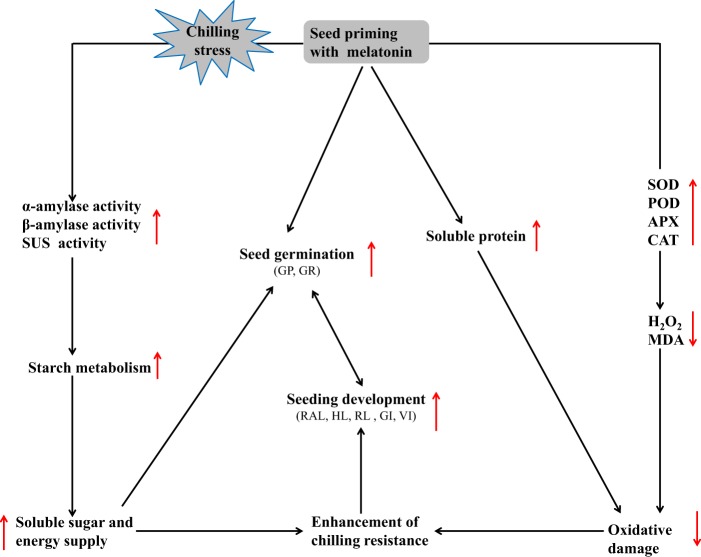
Functional mechanism of seed priming with melatonin enhanced the seed germination quality under chilling stress of waxy corn. The upward arrow indicates an enhancement effect or positive correlation, the downward arrow indicates a reduced effect or negative effect.

In summary, waxy corn seeds primed with MT not only exhibited enhanced GP and GR but also significantly improved germination qualities, as indicated by increased longer radicle length, root length and VI compared with those of the control seedings under CS, while priming time had no significant effect. The physiological effects of MT pretreatment on the improvement of seed tolerance to chilling stress during germination were associated with (1) lower H_2_O_2_ and MDA levels; and significantly increased seed vigour indexes in waxy corn seedlings; (2) osmotic regulation such as assimilation of soluble sugars and proteins for cellular turgor maintenance; (3) increased starch metabolism; as well as activities of related enzymes; and (4) enhanced antioxidant enzyme activities in MT-priming treated seeds of waxy corn under chilling stress (Fig. 8). Thus, seed priming with MT might be a feasible approach to improve waxy maize seed germination under chilling stress.

## Materials and Methods

### Experimental materials and seed priming

Seeds of the waxy corn cultivar Jinongnuo 112 used for this study were obtained from the Maize Research Institute, Jilin Academy of Agricultural Science, China. The seeds were surface sterilized for 10 min in 0.1% sodium hypochlorite, rinsed five times with distilled water, and then soaked in 50 or 100 µM MT solutions. The seeds were primed at 25 °C in the dark for 12 or 24 h under constant gentle agitation. The ratio of seed weight to solution volume (w/v) was 1:5; untreated seeds served as controls. After 12 or 24 h, the primed seeds were immersed in distilled water for 2 min, followed by surface drying with blotting paper, after which they were transferred to an air-drying oven at 30 ± 0.5 °C for 48 h to reduce the moisture content to <14%.

### Experimental design and seed germination

Seeds in each concentrationsof MT priming treatment were randomly selected and evenly placed in culture dishes. Each culture dish was sealed with a sealing membrane to prevent moisture evaporation, and the experiment was set up as a randomized complete block design, with four replicates each treatment.

Half of the dishes were placed in a growth chamber for germination under constant 13 °C for CS, and the remaining dishes were cultured at 25 °C in another growth chamber as a control (NS). The inside of both of the chambers was kept dark for the first three days, but beginning on the fourth day, the chambers provided photosynthetically active radiation at a photon flux density of 350 μmol m^−2^ s^−1^ under a 16/8 h light/dark photoperiod.

### Sampling and seedling characteristics determination

The geminated seeds were counted daily for 9 days. The germination potential (GP, the percentage of normal germinated seeds out of all tested seeds on the 4th day) and germination rate (GR, the percentage of normal germinated seeds out of all tested seeds at the end of the full test) were then calculated on days 4 and 9, respectively.

To study the physiological and biochemical parameters response to seed priming with MT under NS and CS conditions, approximately 10 g of seed embryo from each treatment under 12 h of priming were sampled; immediately frozen in liquid nitrogen; and then stored at −80 °C at 0, 3 and 5 days after germination for biochemical and physiological measurements. Moreover, the same amount of seeds was sampled and dried at 80 °C for 24 h for starch, total suable sugar, sucrose, and reduced sugar content determination.

In addition, after 9 days of germination, seeding morphological indexes, such as hypocotyl length (HL), radicle length (RAL), and root length (RL) were manually obtained with a ruler. Root and shoot dry weights were determined after drying the samples at 80 °C for 24 h. In addition, a germination index (GI) and seed vigour index (VI) were calculated according to the formulae: GI = ∑(Gt/Tt) and VI = GI × seedling dry weight germination, where Gt is the number of germinated seeds on day t, and Tt is the time corresponding to Gt on days.

### H_2_O_2_, MDA and soluble protein content determination

The H_2_O_2_ content was determined according to the method described by Patterson *et al*.^33^. The MDA was tested by an MDA assay kit (Jiancheng Biotech Company, Nanjing China), and the protein content was determined by the Coomassie brilliant blue (CBB) method according to the description of Bradford^34^.

### Assays of antioxidant enzyme activity

The activities of SOD, POD, and CAT were determined by the nitro blue tetrazoliume method, guaiacol method and ultraviolet colourimetry method using detection Kits (A00-1, A00-7, A084-3, Jiancheng Bioengineering Institute, Nanjing, Jiangsu province, China), respectively, and the units of SOD, POD and CAT activity were presented as units per gram of FW.

APX activity was determined by AsA oxidation according to the methods of Mishra *et al*.^35^. Crushed frozen samples (1.0 g) were homogenized with 10 ml of 50 mM phosphate buffer (pH 7.8). Afterwards, 10 ml of the homogenate was centrifuged at 4000 x g for 20 min at 4 °C.The supernatant extract (100 µL) was subsequently added to 2700 µL of PBS (25 mM, pH 7.0), 100 µL of aspartate (7.5 mM) and 100 µL H_2_O_2_ (300 mM), after which the mixture was shaken well and the kinetic activity of A290 was determined in quartz colourimetric cuvettes.

### Extraction of sugars and determination of carbohydrate metabolism

The extraction of sugars was performed using the method described by Hussain *et al*.^1^.Briefly, 0.05 g pulverized samples and 4 mL of 80% ethanol were rapidly added to a graduated test tube with a stopper, which was subsequently transferred to 80 °C water bath under continuous stirring for 40 min after sealing. The tube was then centrifuged at 2000 × g for 2 min, after which the supernatant was collected. Two millilitres of 80% ethanol was then added to the residue for 2 additional extract ions, and the supernatant was combined. Afterward, 10 mg of activated carbon was added to the supernatant fluid, coloured in an 80 °C water bath for 30 min, and then diluted with distilled water in a 10 mL volumetric flask. The filtrate after filtration constituted the sugar solution extract. The total soluble sugar content was determined from the supernatant using the anthrone colourimetry method. Reducing sugars and the sucrose content were estimated from the same filtrate following the method of Zhang^36^. For starch quantification, the procedure was conducted in according to the method descripted by Li^37^ and was determined in accordance with the Risk Assessment Lab of Agri-products Quality and Safety (Changchun), Ministry of Agriculture and Rural Affairs of P.R.C.

### Determination of enzyme activity associated with sugar metabolism

The activities of *α*-amylase, *β*-amylase and sucrose synthetase (SUS) were determined according to the instructions of “α/βamylase (AMS) detection kit” and “SUS detection kit” (Nanjing Jiancheng Bioengineering Institute, China). The units of amylase activity were defined as units per gram of fresh weight; One unit of amylase was presented as per milligram of protein in the sample reaction with substrate for 30 min at 37 °C, and the hydrolysis of 10 mg starch presented a unit of AMS activity.

### Statistical analysis

The data were statistically analysed using the statistical software SPSS package 21.0 (IBM Corp, Armonk, NY, USA). All the data were first test for homogeneity of variance by Levene tests. Seed germination morphological characteristics and chemical properties were analysed by three-way analysis of variance (ANOVA), with ambient temperature (T), MT concentration (0, 50 and 100 µM), priming time (12 and 24 h) and germination days as factors. Duncan’s multiple range test was then applied to determine significant differences.

## Supplementary information

Supplementary information
