# Supplementary material for: Seed Priming with Melatonin Improves the Seed Germination of Waxy Maize under Chilling Stress via Promoting the Antioxidant System and Starch Metabolism
Source: Sci Rep. 2019 Oct 21;9:15044. doi: 10.1038/s41598-019-51122-y (PMC6803654; doi:10.1038/s41598-019-51122-y)
Supplement: Supplementary file 1 — Supplementary information [file 41598_2019_51122_MOESM1_ESM.pdf]

Title:

Seed Priming with Melatonin Improves the Seed Germination of Waxy Maize under Chilling Stress via Promoting the Antioxidant System and Starch Metabolism

Author list:

Qingjun Cao[1],

Gang Li[1*],

Zhengguo Cui[2],

Fentuan Yang[1],

Xiaoli Jiang[1],

Lamine Diallo[2],

Fanli Kong[1]

[1]Jilin Academy of Agriculture Science/Key Laboratory of Northeast crop physiology ecology and cultivation, Ministry of Agriculture in People's Republic of China, Changchun 130033, P.R. China.

[2] College of plant science, Jilin University, Changchun 130062, P.R China.

Germination rate

Stress	Priming time(h)	MT concentration (μM)	experimental replication	erminated seeds,days after germination(DAI						Test seed number in culture dish		Germination rate,days after germination(DAE)					
				4d	5d	6d	7d	8d	9d			4d	5d	6d	7d	8d	9d
CS	12	0	data_1	3	7	8	10	11	11	18		16.67	38.89	44.44	55.56	61.11	61.11
	12	0	data_1	4	9	10	11	12	12	20		20.00	45.00	50.00	55.00	60.00	60.00
	12	0	data_1	3	7	9	10	10	11	18		16.67	38.89	50.00	55.56	55.56	61.11
	12	0	data_1	4	4	6	7	9	10	19		21.05	21.05	31.58	36.84	47.37	52.63
	12	50	data_1	5	8	9	10	12	14	18		27.78	44.44	50.00	55.56	66.67	77.78
	12	50	data_1	6	7	8	10	15	15	20		30.00	35.00	40.00	50.00	75.00	75.00
	12	50	data_1	5	8	9	10	12	13	19		26.32	42.11	47.37	52.63	63.16	68.42
	12	50	data_1	6	8	11	15	16	17	22		27.27	36.36	50.00	68.18	72.73	77.27
	12	100	data_1	5	7	8	14	15	15	17		29.41	41.18	47.06	82.35	88.24	88.24
	12	100	data_1	6	9	11	12	13	15	18		33.33	50.00	61.11	66.67	72.22	83.33
	12	100	data_1	6	9	11	13	14	15	19		31.58	47.37	57.89	68.42	73.68	78.95
	12	100	data_1	7	9	10	12	16	18	22		31.82	40.91	45.45	54.55	72.73	81.82
	24	0	data_1	6	5	9	10	12	14	23		26.09	21.74	39.13	43.48	52.17	60.87
	24	0	data_1	5	3	8	11	13	14	22		22.73	13.64	36.36	50.00	59.09	63.64
	24	0	data_1	4	7	8	10	12	13	20		20.00	35.00	40.00	50.00	60.00	65.00
	24	0	data_1	5	6	8	14	15	15	22		22.73	27.27	36.36	63.64	68.18	68.18
	24	50	data_1	4	6	12	14	16	17	22		18.18	27.27	54.55	63.64	72.73	77.27
	24	50	data_1	6	7	12	15	15	17	22		27.27	31.82	54.55	68.18	68.18	77.27
	24	50	data_1	6	9	12	13	15	17	21		28.57	42.86	57.14	61.90	71.43	80.95
	24	50	data_1	5	8	10	13	15	16	20		25.00	40.00	50.00	65.00	75.00	80.00
	24	100	data_1	7	9	12	15	18	19	22		31.82	40.91	54.55	68.18	81.82	86.36
	24	100	data_1	7	8	13	16	17	19	23		30.43	34.78	56.52	69.57	73.91	82.61
	24	100	data_1	7	8	13	14	17	19	21		33.33	38.10	61.90	66.67	80.95	90.48
	24	100	data_1	8	9	14	16	18	20	23		34.78	39.13	60.87	69.57	78.26	86.96
	12	0	data_1	9	18	21	23	24	24	25		36.00	72.00	84.00	92.00	96.00	96.00

NS	12	0	data_1	10	18	22	23	23	23	24	41.67	75.00	91.67	95.83	95.83	95.83
	12	0	data_1	11	16	21	23	23	23	25	44.00	64.00	84.00	92.00	92.00	92.00
	12	0	data_1	12	19	21	22	24	24	25	48.00	76.00	84.00	88.00	96.00	96.00
	12	50	data_1	11	18	23	25	25	25	25	44.00	72.00	92.00	100.00	100.00	100.00
	12	50	data_1	12	19	24	25	25	25	25	48.00	76.00	96.00	100.00	100.00	100.00
	12	50	data_1	12	20	22	24	24	24	25	48.00	80.00	88.00	96.00	96.00	96.00
	12	50	data_1	11	20	22	23	24	24	24	45.83	83.33	91.67	95.83	100.00	100.00
	12	100	data_1	12	19	22	24	24	24	25	48.00	76.00	88.00	96.00	96.00	96.00
	12	100	data_1	12	18	21	23	23	25	25	48.00	72.00	84.00	92.00	92.00	100.00
	12	100	data_1	12	18	22	23	25	25	25	48.00	72.00	88.00	92.00	100.00	100.00
	12	100	data_1	13	19	22	24	24	24	25	52.00	76.00	88.00	96.00	96.00	96.00
	24	0	data_1	12	14	19	21	22	24	25	48.00	56.00	76.00	84.00	88.00	1.00
	24	0	data_1	11	15	21	23	23	23	24	45.83	62.50	87.50	95.83	95.83	95.83
	24	0	data_1	11	18	20	22	24	24	24	45.83	75.00	83.33	91.67	100.00	100.00
	24	0	data_1	12	19	22	23	23	23	25	48.00	76.00	88.00	92.00	92.00	92.00
	24	50	data_1	13	16	20	23	24	24	25	52.00	64.00	80.00	92.00	96.00	96.00
	24	50	data_1	12	17	21	24	24	25	25	48.00	68.00	84.00	96.00	96.00	100.00
	24	50	data_1	12	19	22	23	25	25	25	48.00	76.00	88.00	92.00	100.00	100.00
	24	50	data_1	11	18	20	23	24	24	25	44.00	72.00	80.00	92.00	96.00	96.00
	24	100	data_1	12	17	19	22	24	24	24	50.00	70.83	79.17	91.67	100.00	100.00
	24	100	data_1	11	18	23	24	24	24	24	45.83	75.00	95.83	100.00	100.00	100.00
	24	100	data_1	13	18	22	22	24	25	25	52.00	72.00	88.00	88.00	96.00	100.00
	24	100	data_1	13	19	21	23	24	24	25	52.00	76.00	84.00	92.00	96.00	96.00

***CS=chilling stress; NS=non-chilling stress**

Germination quality

Stress	Priming time(h)	MT concentration(μM)	experimental replication	Radicle length(mm)	Hypocotyl length(cm)	root length(cm)	Dry weight of germinaton seed(g)	GI	VI
	12	0	data_1	6.9	8.8	7.7	0.37	9.644	3.568
	12	0	data_1	5.5	5.3	6.2	0.35	9.132	3.196
	12	0	data_1	5.1	6.4	4.2	0.3	8.219	2.466
	12	0	data_1	3.7	4.6	4.2	0.41	6.953	2.851
	12	50	data_1	18.5	14.5	11.9	0.45	10.029	4.513
	12	50	data_1	9.6	7.9	9.3	0.44	11.315	4.978
	12	50	data_1	10.3	10	9.5	0.4	10.612	4.245
	12	50	data_1	14.1	10.54	13.6	0.41	12.360	5.068
CS	12	100	data_1	17.3	16.4	16.8	0.35	10.245	3.586
	12	100	data_1	25.1	15.2	11.6	0.39	11.917	4.648
	12	100	data_1	20	12.9	13.2	0.34	13.296	4.521
	12	100	data_1	20.6	8.5	9.4	0.39	13.709	5.346
	24	0	data_1	6.1	4.9	6.3	0.38	8.911	3.386
	24	0	data_1	7.6	9.1	7.9	0.45	8.011	1.762
	24	0	data_1	7.1	4.4	7.34	0.37	9.144	3.118
	24	0	data_1	6.7	7.89	6.8	0.44	7.958	3.343
	24	50	data_1	12.5	6.4	12.5	0.27	12.363	3.832
	24	50	data_1	10.1	16.4	12	0.36	12.349	4.199
	24	50	data_1	18.4	8.1	14.7	0.39	13.463	6.731
	24	50	data_1	16.6	6.4	7.6	0.38	13.013	4.815
	24	100	data_1	20	17.1	13.5	0.28	14.554	3.784
	24	100	data_1	17.1	13	14.5	0.3	14.900	4.917
	24	100	data_1	18.3	10.4	9.4	0.38	12.859	4.115
	24	100	data_1	23	13.1	12.2	0.34	13.674	6.085
	12	0	data_1	54.18	39.82	39.4	0.39	20.538	5.967
	12	0	data_1	46.25	36.85	45.12	0.31	20.695	6.556

	12	0	data_1	41.17	39.65	40.89	0.32	20.388	5.939
	12	0	data_1	45.18	32.1	42.5	0.35	21.479	5.426
	12	50	data_1	58.41	47.45	41.51	0.41	21.324	8.545
	12	50	data_1	52.01	42.15	48.9	0.36	22.608	7.871
	12	50	data_1	missing	34.44	40.12	0.29	22.012	6.813
	12	50	data_1	52.11	42.65	42.25	0.31	22.369	7.309
	12	100	data_1	49.32	47.26	48.51	0.45	21.562	9.260
NS	12	100	data_1	58.49	44.15	42.25	0.35	21.038	9.050
	12	100	data_1	55.17	53.24	48.32	0.32	22.955	8.237
	12	100	data_1	64.39	37.11	46.25	0.34	22.312	8.787
	24	0	data_1	43.28	30.25	42.27	0.36	19.419	7.575
	24	0	data_1	47.39	35.42	36.55	0.32	21.512	6.848
	24	0	data_1	51.22	42.97	39.25	0.38	21.112	8.268
	24	0	data_1	50.15	39.15	45.33	0.35	21.822	7.740
	24	50	data_1	50.25	36.35	46.8	0.34	21.486	7.883
	24	50	data_1	48.48	39.43	42.5	0.32	23.440	7.534
	24	50	data_1	53.02	43.35	39.5	0.36	22.905	8.604
	24	50	data_1	59.07	44.19	40.2	0.35	17.360	8.490
	24	100	data_1	55.02	47.5	48.95	0.41	21.960	abnormal value
	24	100	data_1	52.63	39.7	44.25	0.34	23.362	8.734
	24	100	data_1	55.24	42.2	46.85	0.39	23.771	10.157
	24	100	data_1	50.89	46.3	45.37	0.41	22.600	9.241

***CS=chilling stress; NS=non-chilling stress**

**** the data recored on the 9th day of germination.**

H_2O_2, MDA ,Free prolione and soluble protein content

Stress	Days after germination	MT concentration(μM)	H_2O_2（μmole g-1 FW)				MDA（nmole g-1 FW)				Soluble protein content(mg g^{-1} FW)		
			data_1	data_2	data_3		data_1	data_2	data_3		data_1	data_2	data_3
control		0	6.85	7.26	7.54		7.65	8.32	7.41		12.35	11.41	12.47
	0	50	6.95	7.54	7.42		7.62	7.24	7.47		13.15	12.47	13.13
		100	6.95	6.75	7.11		7.34	6.97	7.71		12.55	13.127	13.01
NS		0	9.49	8.98	9.41		7.18	7.31	6.93		14.45	15.51	16.47
	3	50	9.05	9.11	9.25		7.76	7.54	7.68		16.12	16.4	17.11
		100	7.95	8.22	7.84		7.5	7.41	7.56		17.21	16.07	16.25
		0	8.17	8.58	8.52		7.78	8.15	8.05		23.87	29.11	27.58
	5	50	7.35	7.51	8.02		7.53	8.21	7.65		37.49	32.52	34.71
		100	7.46	7.55	7.65		7.64	7.69	7.81		39.52	40.01	37.65
CS		0	14.05	13.26	13.24		9.58	10.35	9.83		17.35	15.41	16.47
	3	50	10.15	9.54	9.45		9.36	9.51	9.18		19.52	18.61	20.02
		100	7.45	8.35	8.51		8.87	8.89	8.76		20.55	21.127	19.91
		0	15.13	17.22	16.11		9.58	9.65	10.13		25.51	26.28	31.26
	5	50	9.32	9.21	10.25		8.93	9.12	9.18		35.62	33.63	32.57
		100	8.86	9.75	9.35		8.77	8.09	8.16		40.52	35.76	38.446

Oxidative system

Stress	Days after germination(d)	MT concentration(μM)	SOD（U mg-1 FW)				POD（U mg-1 FW)				**CAT**(Umg^{-1} pro)				APX(μmol g^{-1} pro)		
			data_1	data_2	data_3		data_1	data_2	data_3		data_1	data_2	data_3		data_1	data_2	data_3
control		0	20.43	27.26	28.39		10.13	12.55	13.44		6.36	6.11	7.35		15.21	14.55	17.44
	0	50	25.32	30.21	25.44		15.37	12.11	10.32		6.64	6.36	7.56		16.35	20.01	15.12
		100	22.07	30.55	32.21		13.07	14.23	12.81		6.17	6.86	6.41		18.07	20.23	16.81
NS		0	54.53	61.14	57.34		24.35	19.374	22.07		10.55	9.88	10.12		12.15	12.34	13.17
	3	50	65.32	62.71	70.45		25.31	22.35	19.11		14.35	13.68	14.11		14.58	16.32	15.44
		100	69.3	70.15	62.85		30.24	24.94	22		13.25	14.75	13.63		16.55	17.39	17.02
		0	74.55	71.15	67.44		32.11	34.59	39.62		8.52	7.38	8.05		20.11	19.32	18.85
	5	50	80.32	78.11	75.42		38.07	41.17	42.89		12.24	12.34	11.52		22.44	23.55	22.07
		100	79.37	80.11	82.31		40.11	45.39	39.88		13.13	14	12.89		23.57	24.31	22.95
CS		0	110.3	107.25	118.3		49.23	51.05	47.22		13.23	14.28	15.15		19.03	18.41	15.22
	3	50	132.4	152.34	139.5		67.41	61.74	59.35		19.21	19.58	18.25		25.37	24.64	19.33
		100	149.5	151.38	165.6		59.37	63.31	62.22		22.37	20.31	23.21		29.11	27.36	32.28
		0	110.3	107.25	118.3		40.23	39.05	40.22		15.15	16.64	14.11		24.03	26.44	25.22
	5	50	132.4	152.34	139.5		48.41	48.74	49.35		19.25	19.95	21.35		32.27	34.01	32.33
		100	149.5	151.38	165.6		53.37	51.31	52.22		22.37	25.31	23.21		39.15	37.28	33.08

Carbohydrate metabolism

Stress	Days after germination	MT concentratio n(μM)	Starch （mg g-1 FW)			Sucrose （mg g-1 FW)			Reducing sugar （mg g-1 FW)			otal soluble sugars （mg g-1 F		
			data_1	data_2	data_3	data_1	data_2	data_3	data_1	data_2	data_3	data_1	data_2	data_3
control		0	661.2	658	671.5	2.12	1.74	1.85	5.01	6.27	5.42	7.31	6.47	7.28
	0	50	653.1	666.3	657.2	1.92	2.05	2.1	5.65	5.51	6.05	6.35	7.41	7.15
		100	657.3	654.5	668.5	2.13	1.85	2.15	6.07	5.23	5.81	7.07	6.23	7.81
NS		0	615.21	620.3	611.4	3.65	3.47	3.69	8.32	7.95	8.09	12.25	12.2	11.57
	3	50	617.35	614.3	610.1	3.85	3.95	3.53	8.69	8.01	8.12	14.94	15.85	15.68
		100	610.29	615.4	616	3.67	3.88	3.91	8.42	8.09	8.14	15.25	16.73	16.52
		0	562.3	565.4	560.1	2.99	3.05	3.08	9.35	9.22	8.97	13.32	12.57	13.22
	5	50	563.27	556.5	549.2	3.42	3.85	3.67	9.19	9.11	9.32	14.15	14.28	13.65
		100	557.52	550.5	548.6	3.55	3.62	3.51	9.28	9.75	9.62	14.07	15.22	14.17
CS		0	618.2	627.7	625.4	3.31	3.27	3.25	7.33	6.41	7.42	11.31	10.47	13.45
	3	50	635.35	642.1	642.5	3.15	2.984	3.03	7.95	8.27	8.25	16.35	14.41	15.15
		100	643.2	633.8	630.1	3.07	3.03	2.71	8.17	9.03	7.95	16.07	18.23	15.81
		0	582.3	575.5	577.4	2.85	2.71	2.48	8.31	8.42	8.32	12.31	11.47	12.45
	5	50	564.2	552.3	560.1	2.65	2.53	2.35	8.75	9.47	9.25	13.35	14.41	13.15
		100	545.8	538.6	547.9	2.27	2.63	2.41	10.37	9.13	8.98	15.12	14.11	15.81

Enzymatic activity

Stress	Days after germination	MT concentration(μM)	α-amylase activity（U mg^{-1} pro)				-amylase activity（U mg-1 pr				SUS activity（**U mg^{-1} Pro)**		
			data_1	data_2	data_3		data_1	data_2	data_3		data_1	data_2	data_3
control		0	0.36	0.311	0.305		2.43	2.26	2.39		0.93	0.86	0.79
	0	50	0.34	0.316	0.256		2.32	3.21	2.24		0.82	0.901	0.881
		100	0.317	0.386	0.241		2.07	3.15	2.21		0.87	0.95	0.911
NS		0	0.592	0.582	0.621		5.25	5.17	5.45		1.53	1.44	1.53
	3	50	0.631	0.642	0.593		5.33	5.51	5.08		1.54	1.58	1.61
		100	0.631	0.658	0.565		5.24	5.55	5.29		1.49	1.55	1.6
		0	0.596	0.668	0.674		6.78	6.95	7.01		1.63	1.69	1.73
	5	50	0.677	0.683	0.739		6.88	6.49	7.11		1.74	1.71	1.69
		100	0.711	0.645	0.632		7.15	7.09	6.88		1.79	1.65	1.81
CS		0	0.423	0.528	0.465		4.05	4.15	4.44		1.05	1.15	1.04
	3	50	0.621	0.6358	0.625		5.32	4.51	5.22		1.32	1.51	1.22
		100	0.537	0.631	0.621		5.37	4.77	5.21		1.37	1.47	1.51
		0	0.513	0.528	0.515		4.83	5.25	6.034		1.43	1.25	1.034
	5	50	0.521	0.558	0.625		6.41	6.34	6.45		1.41	1.34	1.45
		100	0.587	0.611	0.601		6.47	6.58	6.55		1.47	1.58	1.55
